# Display of a novel carboxylesterase CarCby on *Escherichia coli* cell surface for carbaryl pesticide bioremediation

**DOI:** 10.1186/s12934-022-01821-5

**Published:** 2022-05-28

**Authors:** Yan Liu, Xiaoliang Wang, Sujin Nong, Zehui Bai, Nanyu Han, Qian Wu, Zunxi Huang, Junmei Ding

**Affiliations:** Engineering Research Center of Sustainable Development and Utilization of Biomass Energy, Ministry of Education, Yunnan Normal University, Kunming, 650500 Yunnan China

**Keywords:** Surface display, Carbaryl pesticide, Whole cell biocatalyst, Carboxylesterase, Bioremediation

## Abstract

**Background:**

Carbamate pesticides have been widely used in agricultural and forestry pest control. The large-scale use of carbamates has caused severe toxicity in various systems because of their toxic environmental residues. Carbaryl is a representative carbamate pesticide and hydrolase/carboxylesterase is the initial and critical enzyme for its degradation. Whole-cell biocatalysts have become a powerful tool for environmental bioremediation. Here, a whole cell biocatalyst was constructed by displaying a novel carboxylesterase/hydrolase on the surface of *Escherichia coli* cells for carbaryl bioremediation.

**Results:**

The *carCby* gene, encoding a protein with carbaryl hydrolysis activity was cloned and characterized. Subsequently, CarCby was displayed on the outer membrane of *E. coli* BL21(DE3) cells using the N-terminus of ice nucleation protein as an anchor. The surface localization of CarCby was confirmed by SDS–PAGE and fluorescence microscopy. The optimal temperature and pH of the engineered *E. coli* cells were 30 °C and 7.5, respectively, using *p*NPC4 as a substrate. The whole cell biocatalyst exhibited better stability and maintained approximately 8-fold higher specific enzymatic activity than purified CarCby when incubated at 30 °C for 120 h. In addition, ~ 100% and 50% of the original activity was retained when incubated with the whole cell biocatalyst at 4 ℃ and 30 °C for 35 days, respectively. However, the purified CarCby lost almost 100% of its activity when incubated at 30 °C for 134 h or 37 °C for 96 h, respectively. Finally, approximately 30 mg/L of carbaryl was hydrolyzed by 200 U of the engineered *E. coli* cells in 12 h.

**Conclusions:**

Here, a carbaryl hydrolase-containing surface-displayed system was first constructed, and the whole cell biocatalyst displayed better stability and maintained its catalytic activity. This surface-displayed strategy provides a new solution for the cost-efficient bioremediation of carbaryl and could also have the potential to be used to treat other carbamates in environmental bioremediation.

**Supplementary information:**

The online version contains supplementary material available at 10.1186/s12934-022-01821-5.

## Background

Pesticides, including organophosphates (OPs), carbamates, and pyrethroids, play crucial roles in global agricultural protection and in the prevention or control of potentially fatal diseases caused by pests such as dengue and Leishmania [1]. Due to broad-spectrum, highly effective, structurally simpler, and safer than OPs, carbamate pesticides have been extensively used since the 1970 s [2]. They are divided into bicyclic (including carbofuran and carbaryl), monocyclic (including isoprocarb and propoxur), or linear (including oxamyl and aldicarb) carbamate pesticides [3]. However, carbamate pesticides have been distributed in various ecosystems because of their widespread and excessive applications, which has brought severe public concerns regarding public health (for example, disruption of endocrine and various steroid hormones), crop quality, environmental safety, and even the rapid development of insect resistance [3]. Carbaryl (naphthalen-1-yl *N*-methylcarbamate), a representative naphthalene-based carbamate insecticide, has been extensively applied in controlling pests/bugs in agriculture, and has drastically polluted soil and groundwater [4]. As a known cholinesterase inhibitor, carbaryl can severely affect and disrupt the endocrine and neurological systems [2]. Therefore, the removal or detoxification of carbaryl in the environment is essential. The traditional methods used for pesticide bioremediation, such as physico-chemical approaches, are usually expensive and can generate secondary pollutant contamination to the environment [2, 5]. In comparison, microbial biodegradation is more cost-effective and environmentally friendly [6, 7].

Microorganisms can detoxify or mineralize the desired pollutants into less or no toxic compounds, such as H_2_O and CO_2_ without harmful intermediate generation [8, 9]. Carbaryl and other pesticides can be used as sole carbon sources to maintain microbial growth or biodegradation, and enzymes such as hydrolases/carboxylesterases and mono/dioxygenases secreted by microbial cells are responsible for hydrolyzing pollutants during metabolism [3, 10–12]. To date, several carbaryl-degrading microbial strains have been isolated and reported, such as *Pseudomonas* sp. C4/C5/C6 [13], *Pseudomonas* sp. C5pp [14], *Pseudomonas* sp. XWY-1 [15], *Pseudomonas* sp. OXA20 [16], *Rhizobium* sp. AC100 [17], *Pantoea ananatis* Sd-1 [18], *Novosphingobium* sp. KN65.2 [19], *Sphingomonas* sp. CDS-1 [20], *Sphingbium* sp. CFD-1 [21], *Rhizobium* sp. X9 [22], *Agrobacterium* sp. XWY-2 [8], *Bacillus licheniformis* B-1 [23], *Rhodopseudomonas capsulate* [24], and several fungi, such as *Acremonium* sp. [25] and *Xylaria* sp. BNL1 [26]. Carbaryl is hydrolyzed and destroyed by carboxyl ester hydrolases (EC 3.1.1)/carboxylesterases (EC 3.1.1.1), which is the initial and critical step in the carbaryl metabolic pathway [3]. Additionally, the toxicity of carbaryl is mainly caused by the presence of an ester bond between 1-naphthol and N-methyl carbamic [1]. Therefore, it is necessary to investigate and characterize hydrolases/carboxylesterases for the degradation of carbaryl [3]. To date, several carbaryl-catalyzing hydrolases have been reported, including CarH from *Agrobacterium* sp. XWY-2 [8], CehA from *Sphingbium* sp. CFD-1 [21], McbA from *Pseudomonas* sp. XWY-1/C5pp [15, 27], CfdJ from *Novosphingobium* sp. KN65.2 [19], MCD from *Achromobacter* WM111 [28] (Table 1). Therefore, the isolation of new carbaryl-degrading bacterial or fungal strains and the in-depth characterization or engineering of various carbaryl hydrolases/carboxylesterases are of great importance for further efficient carbaryl bioremediation. Additionally, the purification costs, stability, and activities of hydrolases should also be taken into consideration for large-scale applications in practice.

**Table 1:** Characterized hydrolases for carbaryl biodegradation

Hydrolases	Biochemical properties	Sources	References
CarH	pH = 8.0, Temp = 30 °C, MW ~ 72 kDa	*Agrobacterium* sp. XWY-2	[8]
McbA	pH = 7.0, Temp = 40 °C, MW ~ 87 kDa	*Pseudomonas* sp. XWY-1/C5pp	[15, 27]
CehA	pH/Temp Not available, MW ~ 87 kDa	*Pseudomonas extremaustralis*	[16]
CH	pH = 9.0, Temp = 45 °C, MW ~ 82 kDa	*Rhizobium* sp. AC100	[17]
PaCes7	pH = 7.5, Temp = 37 °C, MW ~ 31 kDa	*Pantoea ananatis* Sd-1	[18]
CfdJ	pH/Temp Not available, MW ~ 87 kDa	*Novosphingobium* sp. KN65.2	[19]
CehA	pH = 7.0, Temp = 40 °C, MW ~ 88 kDa	*Sphingbium* sp. CFD-1	[21]

Surface-displayed strategies that allow peptides or target proteins to be located on the outer membrane of the cell surface have been widely used, such as biocatalyst, biodetoxification, biosensors, live vaccines, and peptide library screening for environmental, industrial, or diagnosis purposes [29, 30]. Localization of target proteins on the cell surface could be used as a whole-cell biocatalyst directly because the substrates do not need to be transported across the membrane into the cells [6]. Surface-displayed systems developed using *E. coli* as a host and the N-terminal of ice nucleation protein (INPN) from *Pseudomonas syringae* as an anchor have been successfully applied in environmental bioremediation, such as biodegradation of phthalic acid esters [31], pesticides [32], antibiotics [33, 34], and absorption of mercury ions/lead [35, 36]. To date, no report using a surface-displayed strategy for carbamate biodegradation has been published.

**Fig. 1:**
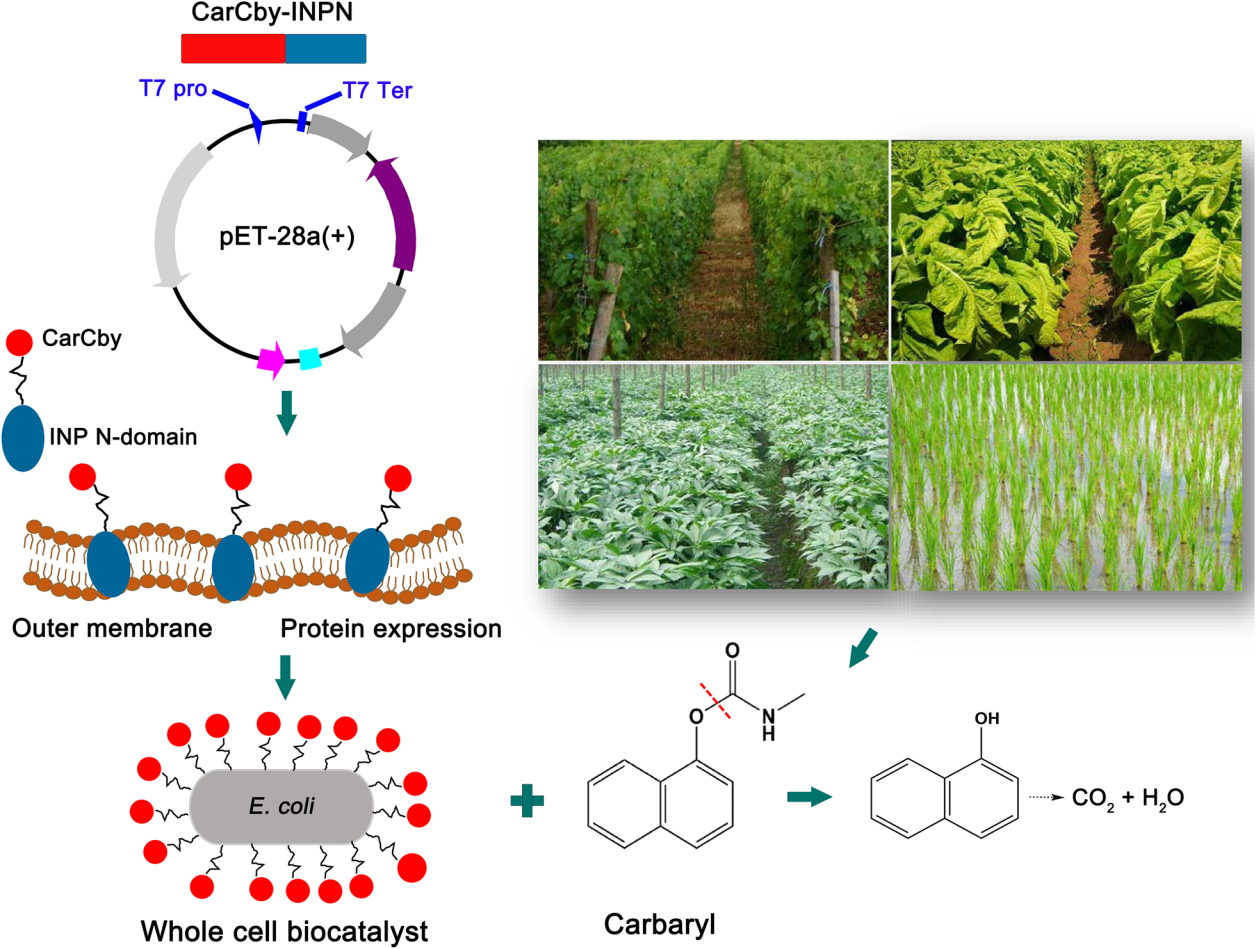
A schematic diagram of CarCby whole-cell biocatalyst construction and its application in carbaryl bioremediation

Here, a novel carboxylesterase gene, *carCby* cloned from *Bacillus velezensis* sd, which can hydrolyze carbaryl, was characterized and further displayed on the surface of *E. coli* cells to function as a whole cell biocatalyst (Fig. 1). The detailed enzymatic characteristics, long-term stability, and carbaryl biodegradation efficiencies of purified CarCby and the whole cell biocatalyst were investigated. This is the first study to identify a carbaryl hydrolase from *B. velezensis* species and a carbaryl-degrading whole cell biocatalyst was constructed which could help to expand the understanding of carbamate degradation.

## Results and discussion

### Sequence analysis of CarCby

Microbial technologies are effective and sustainable for pesticide remediation due to their low cost, survival in extreme environments and complete mineralization. Hydrolysis and oxidation are two major routes of carbamate degradation, and hydrolases/carboxylesterases that are responsible for the hydrolysis process play critical and crucial regulatory roles in the carbamate metabolic pathway [2, 3, 21]. One gene, named here *carCby*, encoding a putative carboxylesterase, was found in the *B. velezensis* sd genome by BLASTP. The ORF of *carCby* (1449 bp) encoded a 482-residue protein with a predicted MW of 52.8 kDa and a theoretical pI of 4.90, and no signal sequence was found. Phylogenetic analysis showed that CarCby clustered and formed an independent clade with a carboxylesterase (WP 069007622.1, 99.79% similarity with CarCby) from *B. velezensis* (Additional file 1: Fig. S1). A BLASTP search against the UniProtKB/Swiss-Port database showed that CarCby shared 63.09% similarity to a para-nitrobenzyl esterase (P37967.2, Query cover 99%) from *B. subtilis* 168, 38.58% identity to a carboxylesterase (P86325.1, Query cover 98%) from *Thermobifida fusca*, 35.69% identity to a pyrethroid hydrolase Ces2e (Q8BK48.1, Query cover 93%) from *Mus musculus*, and 33.66% identity to a phenylcarbamate hydrolase (Q01470.1, Query cover 99%) from *Pseudarthrobacter oxydans*. Multiple sequence alignments using sequences deposited in the Protein Data Bank (PDB) showed that CarCby displayed the highest similarity with an esterase (1QE3, 63.30%, query cover 99%) from *B. subtilis* 168 [37], and moderate identity with carboxylesterase Est55 (2OGS, 41.07%, query cover 98%) from *Geobacillus stearothermophilus* [38] and carboxylic ester hydrolase (5A2G, 34.27%, query cover 97%) from *Hungatella hathewayi* DSM 13,479 [39].

**Fig. 2:**
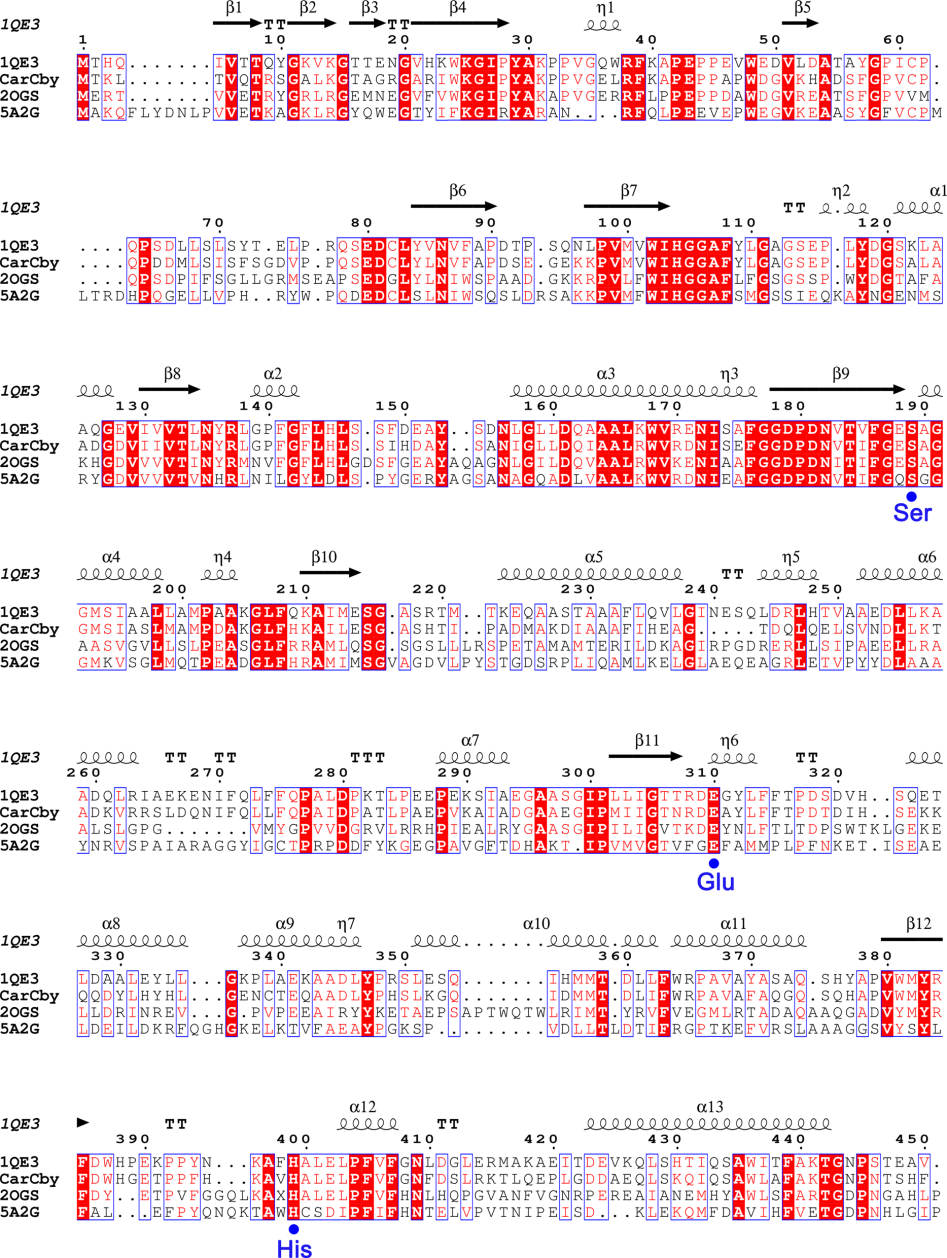
Multiple sequence alignment of CarCby with other structure-based esterases, including an esterase (PDB: 1QE3), Est55 (PDB: 2OGS, a carboxylesterase), and a hydrolase (PDB: 5A2G). The secondary structural elements of the esterase (PDB code: 1QE3) are shown above the aligned sequences. The alignments were prepared using ClustalX2 and ESPript 3.0

Based on the multiple alignments, CarCby possesses a putative catalytic triad Ser^190^-Glu^306^-His^395^, and the nucleophilic Ser located in the conserved motif Gly^188^-X-Ser^190^-X-Gly^192^ (GXSXG) as GESAG in CarCby (Fig. 2). To investigate the catalytic triad, Ser^190^-Glu^306^-His^395^ were separately substituted with alanine (Additional file 2: Fig. S2), and the corrected mutants were confirmed by sequencing. Compared with the wild type CarCby, the mutants lost almost all their enzymatic activities (Additional file 3: Table S1), indicating that Ser^190^-Glu^306^-His^395^ composed a catalytic triad of CarCby. To date, none of the above aligned hydrolases have been characterized or reported for pesticide degradation. Moreover, CarCby exhibited no obvious similarity with characterized carbamate hydrolases, including CarH [8], CehA [21], McbA [15, 27], CfdJ [19], and MCD [28] (Table 1). Therefore, an in-depth investigation of CarCby is necessary and essential.

### Expression of CarCby and CarCby-INPN fusion proteins

**Fig. 3:**
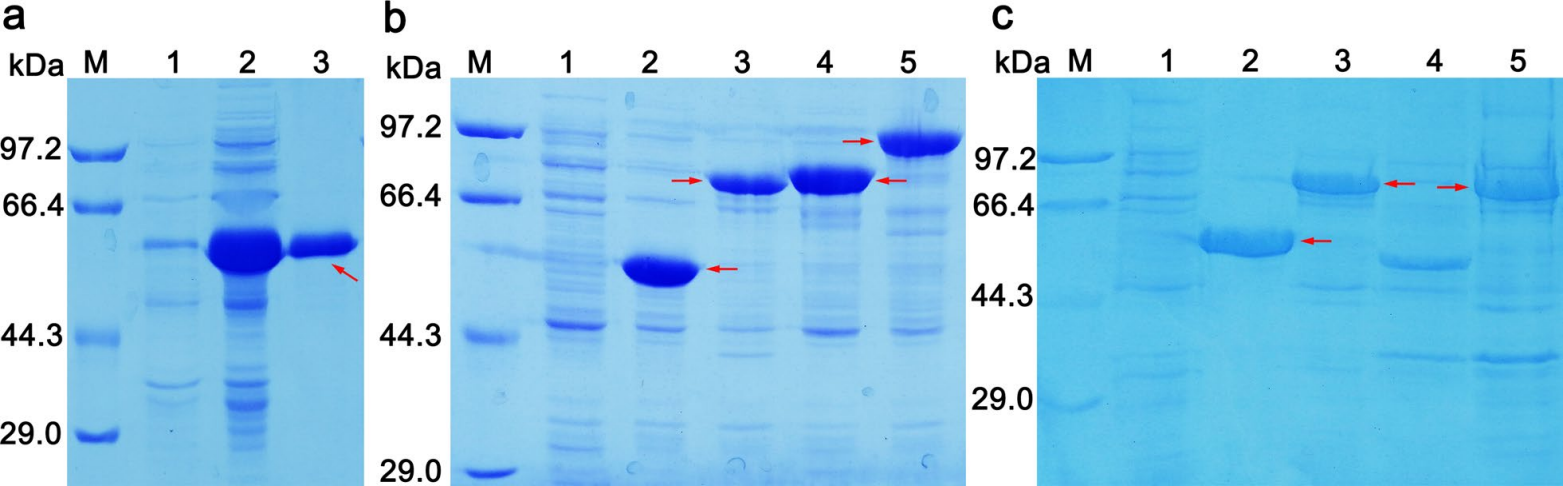
SDS–PAGE analysis. **a** Purification of CarCby. Lane M: protein marker (kDa); Lane 1: supernatant of *E. coli* BL21(DE3)[pET-28a(+)] strain (control) lysates; Lane 2: supernatant of *E. coli* BL21(DE3)[pET-28a(+)/CarCby] strain lysates; Lane 3: purified recombinant CarCby (~ 52 kDa). **b** Expression of CarCby-INPN, CarCby-GFP, and CarCby-INPN-GFP fusion proteins. Lane 1: supernatant of *E. coli* BL21(DE3)[pET-28a(+)] strain (control) lysates; Lane 2: supernatant of *E. coli* BL21(DE3)[pET-28a(+)/CarCby] strain (positive control) lysates; Lane 3, 4, 5: supernatant of *E. coli* BL21(DE3) containing pET-28a(+)*/*CarCby*/*INPN, pET-28a(+)*/*CarCby*/*GFP, and pET-28a(+)*/*CarCby*/*INPN*/*GFP lysates, respectively. **c** Expression of CarCby on the *E. coli* cell surface. Lane 1: supernatant of *E. coli* BL21(DE3) cells containing pET-28a(+) plasmid (control) lysates; Lane 2: purified CarCby; Lane 3, 4, 5: cytoplasmic, inner, and outer membrane fractions of *E. coli* BL21(DE3)[pET-28a(+)*/*CarCby*/*INPN] strain. The recombinant CarCby fusion proteins are marked with red arrows

The purified CarCby-His_6_ protein from *E. coli* BL21(DE3) cells was observed as a single band on 10% SDS–PAGE, with an approximate size that agreed with its theoretical MW calculated from its amino acid sequence (Fig. 3a, lane 3). INPN was used as an anchor to construct CarCby whole cell biocatalyst [31, 32]. To confirm that CarCby-INPN could indeed be located on the outside of the outer membrane, the recombinant plasmid pET-28a(+)*/*CarCby*/*INPN/GFP that used GFP as a reporter was constructed. *E. coli* BL21(DE3) cells harboring pET-28a(+)/CarCby/GFP were used as a control. First, to check the successful expression of CarCby-INPN, CarCby-GFP, and CarCby-INPN-GFP fusion proteins, *E. coli* BL21(DE3) cells containing pET-28a(+)*/*CarCby*/*INPN, pET-28a(+)*/*CarCby*/*GFP, and pET-28a(+)*/*CarCby*/*INPN*/*GFP plasmids were collected after induction and lysed by ultrasonication. Bands corresponding to CarCby-INPN (CarCby, ~ 52 kDa; INPN, ~ 20 kDa; CarCby-INPN, ~ 72 kDa, line 3, red arrow), CarCby-GFP (GFP, ~ 27 kDa; CarCby-GFP, ~ 79 kDa, line 4, red arrow), and CarCby-INPN-GFP (CarCby-INPN-GFP, ~ 99 kDa, line 5, red arrow) were detected that were consistent with their theoretical MW (Fig. 3b). Then, CarCby expression on the *E. coli* cell surface was determined by analyzing subcellular fractions of *E. coli* BL21(DE3)[pET-28a(+)*/*CarCby*/*INPN] cells by SDS–PAGE. A band corresponding to ~ 72 kDa was detected in both the cytoplasmic (Fig. 3c, lane 3) and outer membrane fractions (Fig. 3c, lane 5), and no band at the same position was observed in the inner membrane fraction (Fig. 3c, lane 4). The specific activity of the whole cell biocatalyst was 1036.57 U, while the activity increased to 1453.48 U after thorough lysis by ultrasonication, which indirectly confirmed that CarCby was expressed in both the cytosol and outer membrane of *E. coli* cells. To evaluate the cell integrity of BL21(DE3) cells containing pET-28a(+)*/*CarCby or pET-28a(+)*/*CarCby*/*INPN, BL21(DE3) cells harboring the blank vector pET-28a(+) were used as a control. As shown in Additional file 4: Fig. S3, the absorbance values of BL21(DE3) cells containing pET-28a(+)*/*CarCby or pET-28a(+)*/*CarCby*/*INPN were slightly lower than those of BL21(DE3) cells containing blank vector, while no significant difference was observed between the two engineered strains. The results indicated that the expression of CarCby in the cytoplasm or on the cell surface had no obvious negative effect on the host strain. Additionally, other surface-displayed host cells, such as *E. coli* K-12 and its derivatives, might also be employed due to their better cell envelope composition, such as less membrane permeability and lower amounts of lipoproteins, which can facilitate the assembly of outer membrane β-barrel protein comparable with the *E. coli* B strain [40].

**Fig. 4:**
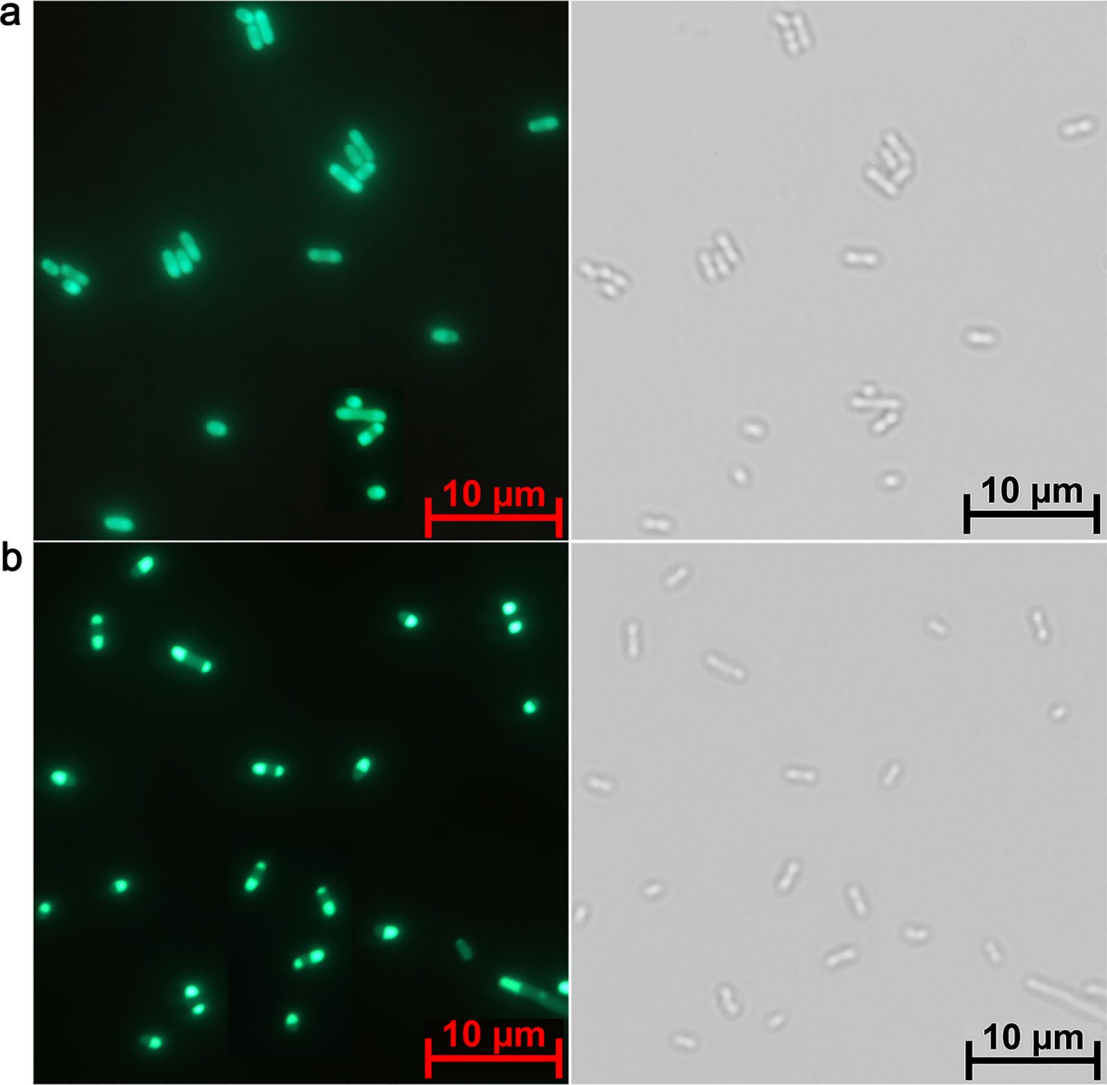
Fluorescence micrographs. **a** and **b** *E. coli* BL21(DE3) cells harboring (**a**) pET-28a(+)*/*CarCby*/*GFP and (**b**) pET-28a(+)*/*CarCby*/*INPN*/*GFP. Left panel, fluorescence microphotographs; Right panel, bright field. The phase contrast and fluorescence images were taken using a Zeiss Axio Imager Z2 (Germany) with a 100 × oil immersion objective

To further confirm that CarCby-INPN was localized on the outer membrane, a fluorescence assay was carried out. The green fluorescence of BL21(DE3) cells containing pET-28a(+)*/*CarCby*/*INPN*/*GFP concentrated at either both poles or the membrane (Fig. 4b, left panel), and it was distributed uniformly in the BL21(DE3)[pET-28a(+)*/*CarCby*/*GFP] strain (Fig. 4a, left panel). The localization of CarCby-INPN also agrees with other work reported previously using the same surface-displayed strategy [31, 32]. All the results suggested that CarCby was displayed and expressed successfully on the *E. coli* cell surface. In rod-shaped bacteria, Govindarajan et al. showed that the poles might provide specialized sites for a wide variety of cellular functions, including the localization of proteins [41]. Therefore, it is speculated that the CarCby-INPN-GFP fusion protein translocated to the outer membrane and concentrated on both poles of *E. coli* cells after it was synthetized in the cytoplasm with the help of INPN. Additionally, a previous study also showed that many proteins that are integral or peripheral to the cytoplasmic membrane are concentrated at the cell poles, such as the lactose permease LacY and osmosensory transporter ProP [42]. As an outer membrane protein, the localization of INPN in *E. coli* cells will be studied in our future work. Furthermore, it is interesting to elucidate the mechanism of protein organization at both poles of bacterial cells and their exact functions, whether or not the poles just serve as a receptacle for proteins, superstructures or membrane domains with no functional effects.

### Characteristics and stability of purified CarCby and surface-displayed CarCby

**Fig. 5:**
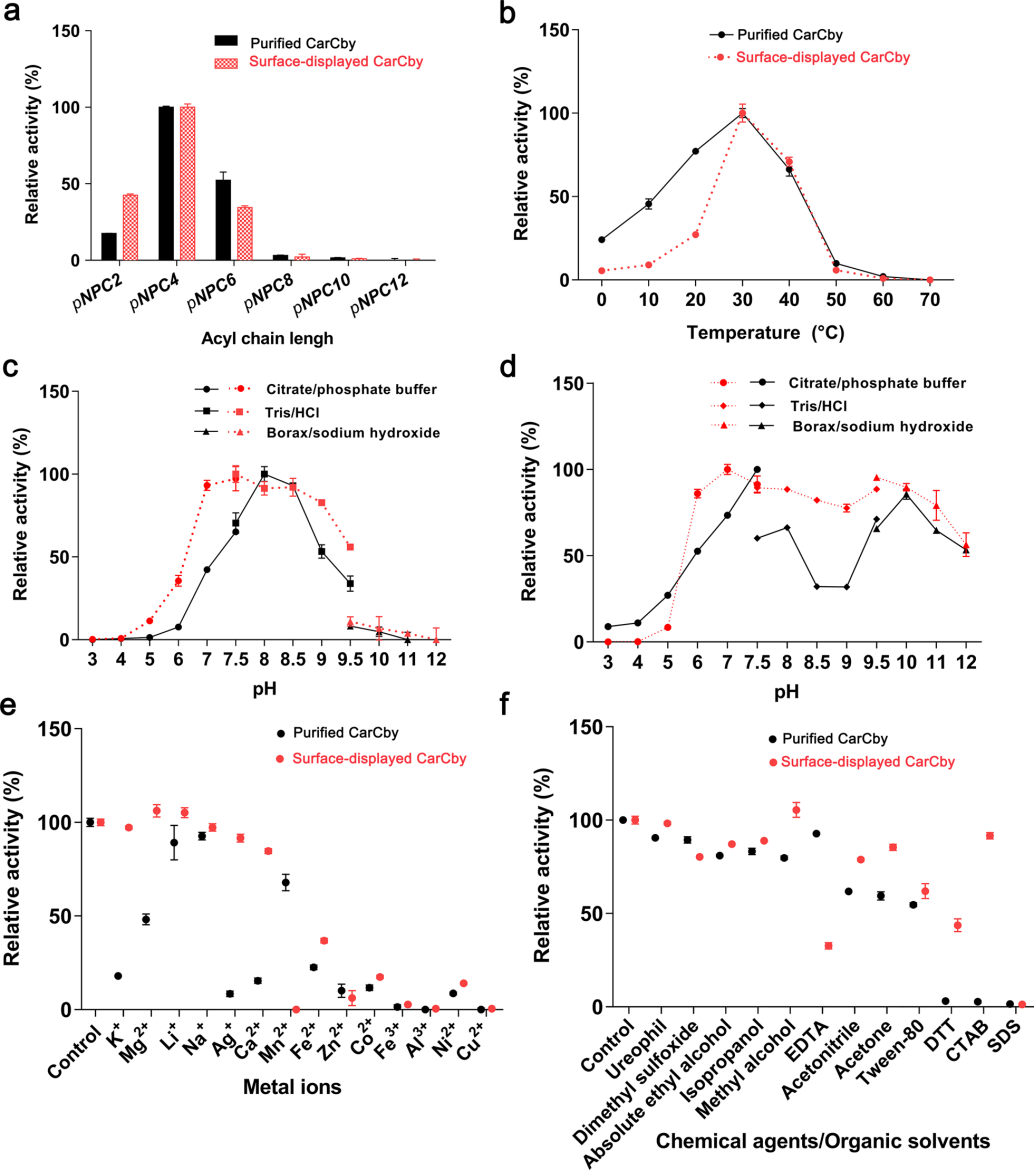
Biochemical characterization of purified CarCby and surface-displayed CarCby. **a** The optimal substrates. **b** Effects of temperature and **c** pH. **d** pH stability. **e** Effects of metal ions and **f** chemical reagents/organic solvents. Red and black lines represent the surface-displayed CarCby and the purified CarCby, respectively

The purified CarCby and surface-displayed CarCby were both active on *p*NPC2 to *p*NPC8, and both displayed the highest activity when using *p*NPC4 as substrate (Fig. 5a). The optimal temperature was investigated between 0 and 70 °C, and the highest activity of purified CarCby and the whole cell biocatalyst was observed at 30 °C; >50% of the original activity was maintained for both between 15 and 40 °C (Fig. 5b). The optimal pH for purified CarCby and the whole cell biocatalyst were pH 8.0 and 7.5 (Fig. 5c), respectively. The pH stability of surface-displayed CarCby (stable between 6.0 and 12.0) was better than that of purified CarCby (stable between 9.5 and 12.0), and > 70% activity of the whole cell biocatalyst was retained after incubation at pH values ranging from 6.0 to 11.0 for 1 h at 37 °C (Fig. 5d), indicating that the whole cell biocatalyst might be more applicable for practical use. Under optimal conditions, the specific activities of purified CarCby and the whole cell biocatalyst (freeze-dried powder) were 1323.9 and 859.38 U/mg, respectively. To maintain long storage, the whole cell biocatalyst was made as a freeze-dried powder. However, a certain percentage of activity might be lost during the process of preparation. Additionally, the *K*_*m*_, *K*_*cat*_, and *K*_*cat*_*/K*_*m*_ of purified CarCby for *p*NPC4 were 0.43 ± 0.11 mM, 14789.0 S^− 1^, 34393.0 S^− 1^ mM^− 1^, respectively.

The effects of metal ions and chemical reagents/organic solvents on free CarCby and surface-displayed CarCby activity were investigated. Mg^2+^, Li^+^, and methyl alcohol activated surface-displayed CarCby, and no obvious influence was detected by K^+^, Na^+^, Ag^+^, and ureophil. Fe^2+^, Zn^2+^, Co^2+^, Fe^3+^, Al^3+^, Ni^2+^, Cu^2+^, and SDS exhibited strong inhibitory effects (80-100% inhibition) on both purified CarCby and the whole cell biocatalyst. Dimethyl sulfoxide, absolute ethyl alcohol, and isopropanol had moderate inhibitory effects (11-20% inhibition) on both. However, Mg^2+^, K^+^, Ag^+^, Ca^2+^, methyl alcohol, acetone, CTAB, and DTT had stronger inhibitory effects on purified CarCby than on surface-displayed CarCby (Fig. 5e and f). The results indicated that the surface-displayed CarCby might be more applicable for practical pollutant remediation due to its better stability and tolerance to many metal ions or chemical reagents.

**Fig. 6:**
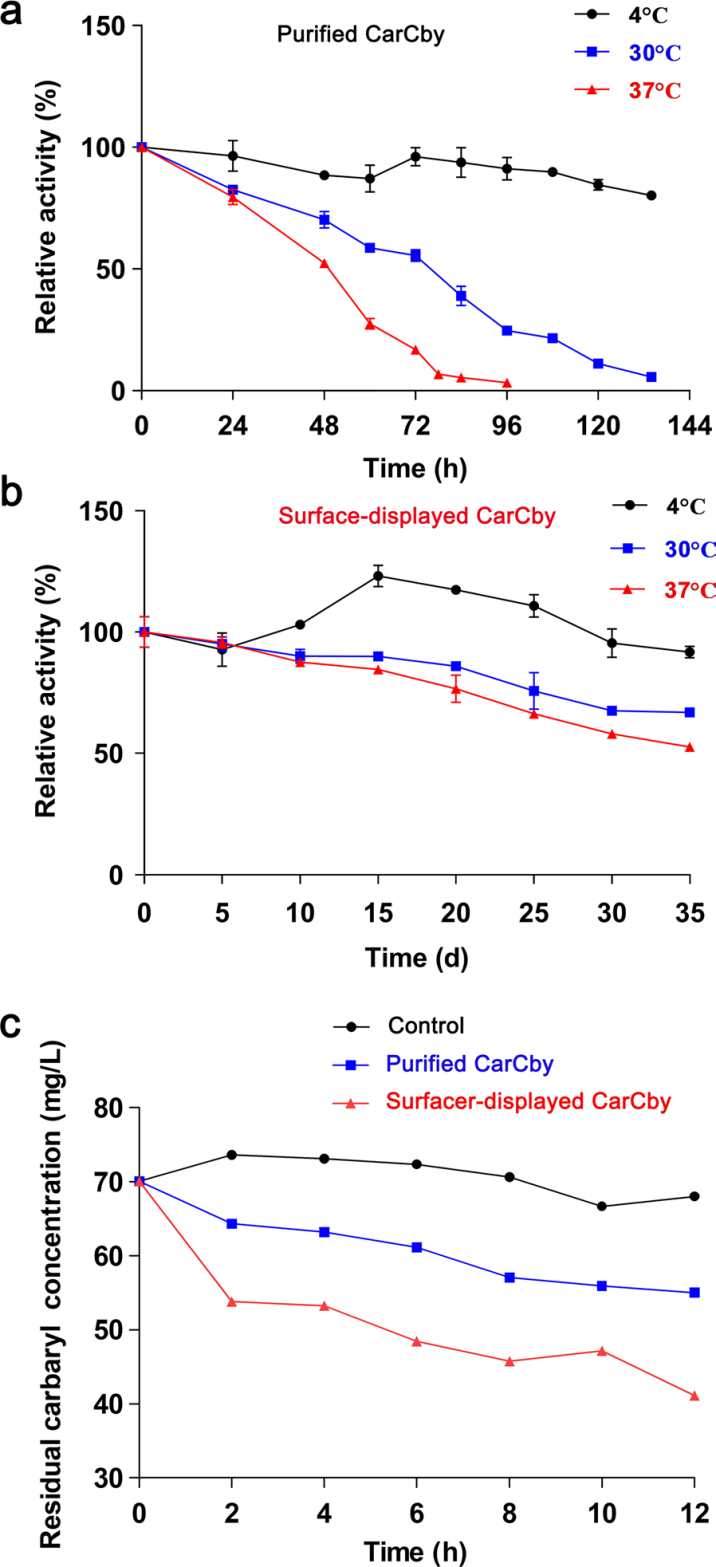
Long-term stability of **a** purified CarCby and **b** surface-displayed CarCby. **c** carbaryl degradation. Values are means ± standard deviations of three replicates

No inhibitory effect was observed for *E. coli* BL21(DE3) cells containing pET-28a(+)*/*CarCby*/*INPN or pET-28a(+)*/*CarCby, and both reached the same cell density at the indicated incubation time. To investigate their stability, both activities were determined regularly at 4 °C, 30 °C, and 37 °C. No enzymatic activity loss of the whole cell biocatalyst was observed when incubated at 4 °C for 35 days (Fig. 6b). Increased enzymatic activity of the whole cell biocatalyst was detected between 10 and 25 days, which might be caused by the release of CarCby residing in the cytoplasmic fraction after cell death and lysis. Similarly, cells also died and lysed when they were incubated at 30 or 37 °C. However, the released intracellular CarCby was not stable at 30 and 37 °C, as observed for free CarCby in Fig. 6a. Therefore, slightly but not sharply decreased activity was detected when the whole cell biocatalyst was incubated at 30 or 37 °C (Fig. 6b).

The same phenomenon was also noticed in a previous report of a surface-displayed organophosphorus hydrolase using the same strategy [43]. Additionally, the engineered bacteria retained > 50% activity after incubation at 30 and 37 °C for 35 days (Fig. 6b). Conversely, the purified CarCby lost almost 100% of its activity when incubated at 30 °C for 134, and 37 °C for 96 h (Fig. 6a). After incubation with 2.5 U of whole cell biocatalyst and purified CarCby at 30 °C for 120 h, approximately 2.365 and 0.292 U of enzymatic activity retained, respectively, that ~ 8-fold higher enzymatic activity of whole cell biocatalyst was maintained than purified CarCby (Fig. 6a and b). Therefore, better stability of surface-displayed CarCby than the purified CarCby was observed. The high reaction selectivity and milder/greener reaction conditions of purified enzymes have made them widely applied in various fields, including industry, environmental treatment, food chemistry, pharmacy, and so on. However, the sensitivity to denaturing agents, environmental parameters, proteases, and inefficient reusage limits the onsite usages of the purified enzymes [44]. Attachment of purified enzymes on a solid matrix can make them more stable, more reusable, and easier to recover [45]. However, preparation processes such as enzyme purification and matrix selection for immobilization are usually time-consuming and expensive [46]. The microbial surface-displayed strategy of fusing target enzymes with cell wall carrier proteins avoids laborious, time-consuming processes of immobilization [47, 48]. Here, the CarCby whole cell biocatalyst presented a potential candidate for the biodegradation of carbaryl, a kind of carbamate pesticide.

### Intermediate product identification and probable metabolic pathway of carbaryl

The degradation of carbaryl (70 mg/L) by purified CarCby and the whole cell biocatalyst was investigated. Approximately 30 mg/L (43%) and 15 mg/L (21%) of the carbaryl was hydrolyzed in 12 h by the whole cell biocatalyst and the purified CarCby, respectively (Fig. 6c). In the control, the same reaction conditions were used but without the addition of purified CarCby or the whole cell biocatalyst. Intermediate products of the carbaryl degradation reaction catalyzed by free CarCby or the whole cell biocatalyst were identified by comparison to the mass spectra (*m/z*). One compound with an *m/z* [M + H]^+^ of 145 was identified as 1-naphthol in both reactions (Additional file 5: Fig. S4), which matched the protonated derivative of 1-naphthol (C_10_H_8_O, 144). Carbaryl, with an *m/z* [M + H]^+^ of 202, was also detected in both reactions as in the control; this finding was consistent with the protonated derivative of carbaryl (C_12_H_11_NO_2_, 201). The results indicated that the main intermediate product of carbaryl was 1-naphthol after catalysis by free CarCby or whole cell biocatalyst. Therefore, the whole cell biocatalyst could be used for carbaryl bioremediation in practical environmental bioremediation due to its higher catalytic efficiency and better stability/availability. In addition, a possible metabolic pathway of carbaryl was proposed (Additional file 6: Fig. S5) based on the predicted proteins located in the genome of *B. velezensis* sd (Additional file 7: Table S2). In nature, there are usually various kinds of pesticides, including organophosphates, carbamates, or pyrethroids, at a single site that might be hydrolyzed by hydrolases/carboxylesterases by cleaving or hydrolyzing their ester bond [3, 49]. Therefore, this bioremediation of pollutants other than carbaryl using surface-displayed CarCby or other identified functional enzymes and the characterization of the predicted enzymes in the carbaryl metabolic pathway in *B. velezensis* sd deserve to be studied in detail in the future.

## Conclusions

This is the first time that a carboxylesterase, CarCby, was functionally displayed on the surface of *E. coli* cells with high activity and long-term stability for carbaryl biodegradation, a kind of carbamate pesticide. Carbaryl (30 mg/L) could be hydrolyzed in 12 h by the engineered bacteria. The results obtained here indicated that the biocatalyst constructed using a surface-displayed strategy provides an efficient approach for pesticide bioremediation with no need for enzyme extraction and purification. Therefore, the exploration and characterization of new/novel functional hydrolases from different microorganisms that can be used to construct more functional whole cell biocatalysts for the bioremediation of various environmental pollutants will also constitute an important aspect of our future work.

## Methods

### Substrates, medium and chemicals

*p*NP acetate (*p*NPC2), *p*NP butyrate (*p*NPC4), *p*NP caproate (*p*NPC6), *p*NP caprylate (*p*NPC8), *p*NP caprate (*p*NPC10), and *p*NP laurate (*p*NPC12) were purchased from Sigma–Aldrich (USA). Carbaryl (99%) and 1-naphthol (99.9%, standard) were obtained from Aladdin Biochemical Technology Co., Ltd. (Shanghai, China) and dissolved in acetonitrile at 1 mg/mL as the working solution. All molecular biological enzymes were purchased from Takara (Dalian, China) or Vazyme (Nanjing, China). HPLC-grade and all other analytical reagents were from Sinopharm (Shanghai, China). Ni-NTA agarose was obtained from Qiagen (Hilden, Germany). Lysogeny broth (LB) medium (pH 7.0, g/L) contained 10.0 g tryptone, 5.0 g yeast extract, and 10.0 g NaCl, and agar was added to a final concentration of 2.0% (w/v) to make solid medium if necessary.

### Bacterial strains and plasmids

*B. velezensis* sd was isolated and characterized previously from farmland where pesticides were used for several years [32]. The strains and plasmids used in this study are shown in Table 2. *B. velezensis* sd and all the *E. coli* strains were grown in LB medium at 37 ℃ or on LB agar plates supplemented with 50 µg/mL kanamycin (Kan^r^) if necessary.

**Table 2:** Strains, plasmids, and primers used in this study

Strains, plasmids, and primers	Description ^a^	Source or reference
Strains		
*B. velezensis* sd	Source of *carCby* gene	This lab
*E. coli* DH5α	clone host	This lab
*E. coli* BL21(DE3)	Expression host	This lab
Plasmids		
pEGFP-N3	Containing *gfp* gene (720 bp), Amp^r^	This lab
pMD18-T/inak	T-clone vector ligated with the synthesized *inak* gene (699 bp) from *Pseudomonas syringae*, Amp^r^	This lab
pMD18-T/inpn	Containing N-terminal of *inak* gene (537 bp), Amp^r^	This lab
pET-28a( +)	Expression vector, Kan^r^	Novagen
pET-28a( +)/CarCby	Expression vector coding for *carCby*	This study
pET-28a( +)/CarCby*/*INPN	Expression vector coding for *carCby* and *INPN*	This study
pET-28a( +)/CarCby*/*GFP	Expression vector coding for *carCby* and *GFP*	This study
pET-28a( +)/CarCby*/*INPN*/*GFP	Expression vector coding for *carCby*, *INPN* and *GFP*	This study
Primers (5' → 3')^a^	Sequences	
P1	AACTTTAAGAAGGAGATATACCatgacaaaacttaccgttcaaa	
P2	TCGACGGAGCTCGAATTCGGATCTGCTTGAAACAGGATACGGCGT	
P3	TCCTGTTTCAAGCAGATCCGAATATGACTCTCGACAAGGCGTTGG	
P4	TGCTCGAGTGCGGCCGCAAGCTTGGTCTGCAAATTCTGCGGC	
P5	TCCTGTTTCAAGCAGATCCGAATATGGTGAGCAAGGGCGAGG	
P6	TGCTCGAGTGCGGCCGCAAGCTTCTTGTACAGCTCGTCCATGCC	
P7	CAGAATTTGCAGACCAAGCTTATGGTGAGCAAGGGCGAGG	
P8	GTGGTGGTGGTGCTCGAGCTTGTACAGCTCGTCCATGCC	
Ser190A-For	ATTTGGTGAA**GCG**GCAGGCGGCATGAGCATCG	
Ser190A-Rev	CTGC**CGC**TTCACCAAATATCGTGACGTTATCC	
Glu306A-For	CAATCGTGAT**GCA**GCATATTTGTTTTTCACCCCTGA	
Glu306A-Rev	ATGC**TGC**ATCACGATTGGTTCCGATGATCATA	
His395A-For	ATAAAGCCGTT**GCC**GCTCTGGAATTGCCGTTTGT	
His395A-Rev	AGCGGCAAC**GGC**TTTATGAAACGGCGGTGTTT	

^a^ The upstream and downstream recombined sequences with plasmids are underlined. The modified codons are shown in bold with red color

### Alignment analysis of CarCby

The amino acid sequence was predicted by the Swiss-Prot database and BlastP based on the genomic sequence of *B. velezensis* sd (PRJNA773980) reported previously [32]. The Carbohydrate-Active Enzymes Database (CAZy) and dbCAN online tools were used to classify CarCby. MEGA7 [50] was used to align CarCby with other reported proteins, and the multiple alignments were rendered by ESPript 3.0 [51]. A phylogenetic tree was constructed using MEGA7 with the neighbor-joining method. SignalP 5.0 (online tool) and ExPASy were employed to predict signal peptides, theoretical molecular mass (MW), and isoelectric point (pI) of CarCby, respectively.

### Plasmid construction

The *carCby* gene was cloned from the genomic DNA of *B. velezensis* sd using P1/P2 primers. After gel purification, the PCR products were ligated into *Bam*HI/*Nco*I digested pET-28a(+) plasmid to generate pET-28a(+)/CarCby. Primers P3/P4 were used to amplify INPN using a previously constructed pMD18-T/inak plasmid as a template [31], and the purified PCR products were ligated into *Eco*RI/*Hin*dIII digested pET-28a(+)/CarCby to generate pET-28a(+)/CarCby/INPN. GFP was cloned from pEGFP-N3 [31] using P5/P6 and P7/P8 primers, and then the PCR products were ligated into *Eco*RI/*Hin*dIII- and *Hin*dIII*/Xho*I-digested pET-28a(+)/CarCby and pET-28a(+)/CarCby/INPN to generate pET-28a(+)/CarCby/GFP and pET-28a(+)/CarCby/INPN/GFP, respectively. All the constructed plasmids were transformed into *E. coli* DH5α competent cells, and correct inserts were confirmed by DNA sequencing. All the primers used are provided in Table 2, and the construction processes of the recombinant plasmids are depicted in Additional file 8: Fig. S6.

### Expression and purification of recombinant CarCby

All the constructed plasmids were separately transformed into *E. coli* BL21(DE3) competent cells and incubated in LB broth (kan^r^, 50 µg/mL) to an OD_600_ of 0.5–0.7 at 37 ℃. Subsequently, isopropyl *β*-D-1-thiogalactopyranoside (IPTG) was added to a final concentration of 0.6 mM, and the cells were continuously cultured for another 18–20 h at 16 ℃. Then, the cells were collected, washed, and disrupted by ultrasonication on ice. The supernatant containing CarCby was further purified using Ni-NTA agarose after centrifugation at 13,000 × *g* for 40 min at 4 ℃. The nontarget protein was washed with 0–80 mM imidazole in 20 mM Tris-HCl (pH 8.0), 10% glycerin (w/v), 50 mM NaCl, and target CarCby-His_6_ was eluted using the same buffer but 500 mM imidazole was used instead. Imidazole was removed with a 10 kDa Amicon ultrafiltration tube. The purity, molecular mass, and concentration of purified CarCby were checked by SDS–PAGE and a BCA Protein Assay Kit from EpiZyme (Shanghai, China).

### Expression and localization of CarCby on the surface of ***E. coli*** cells

*E. coli* BL21(DE3) cells harboring pET-28a(+)/CarCby/INPN plasmid were collected at 4 ℃ (6000 × g, 15 min) after induction. Then, the cells were washed twice with PBS buffer (pH 7.4), and the cytoplasmic and outer/inner fractions were obtained as reported previously [31]. Additionally, the localization of CarCby in BL21(DE3) cells containing pET-28a(+)/CarCby/INPN/GFP was examined by a fluorescence microscope, and the BL21(DE3)[pET-28a(+)/CarCby/GFP] strain was used as the control group [31].

### Measurement of outer membrane integrity

The outer membrane integrity analysis was based on a method described by Zhe et al. [52]. Briefly, the whole cell biocatalysts were collected and washed three times with PBS buffer (pH 7.4) and then diluted with PBS containing 2 mM EDTA to an OD_600_ of 1.0. The change in absorbance at OD_595_ due to cell lysis was recorded every 30 min for 6 h.

### Characteristics of purified CarCby and the whole cell biocatalyst

The standard enzymatic reaction composed of 50 mM Tris-HCl buffer (pH 8.0 for purified CarCby and pH 7.5 for surface-displayed CarCby), 0.6 mM *p*NPC4, and 0.15 µg purified CarCby or whole-cell biocatalyst was incubated at 30 °C, followed by termination with 1 M Na_2_CO_3_. After induction, 1 mL of the engineered bacterial cells was harvested by centrifugation (8,000 rpm, 15 min), washed twice with PBS (pH 7.4) and weighed. Then, 0.15 µg of engineered bacterial cells was harvested according to the above proportion (total weight of whole cell biocatalyst after centrifugation by 1 mL cell culture). To make freeze-dried powder of the whole cell biocatalyst, a method was referenced based on Li et al. reported previously [34]. The amount of *p*NP released was determined at 405 nm continuously, and one enzymatic activity unit (U) was regarded as the amount of enzyme required to release 1 µM of *p*NP per minute. Enzymatic kinetics were determined using various concentrations of *p*NPC4 (0.3 to 2.4 mM) in the reactions. *K*_*m*_, *V*_*max*_, and *K*_*cat*_ were calculated by nonlinear regression fitted to the Michaelis–Menten equation.

Three kinds of buffers (50 mM) including citrate-phosphate buffer (pH 3.0–7.5), Tris-HCl (pH 7.5–9.5), boric acid-NaOH (pH 9.5–12.0) and temperatures between 0 and 70 °C were employed to determine the optimal pH and temperature of purified CarCby and the whole cell biocatalyst, respectively. pH stability was investigated by preincubation with 2.5 U of purified CarCby or whole-cell biocatalyst in various buffers (pH 3.0–12.0) at 37 °C for 1 h. The highest enzymatic activity of both catalysts was regarded as 100%, and the relative activity was determined after various treatments. Long-term stability was studied by incubating 2.5 U of free CarCby and whole cell biocatalyst (freeze-dried powder) in citrate-phosphate buffer (50 mM) under their optimal pH at 4 °C, 30 °C, and 37 °C for approximately one month, and residual relative enzyme activities were determined. The threshold of enzymatic activity was defined as 100%.

The effects of metal ions and chemical agents/organic solvents on both catalysts were determined by adding them to the standard reaction and incubating at 30 °C for 5 min. The residual enzymatic activity was determined, and enzymatic activity without metal ions or chemical agents/organic solvents was regarded as 100%. All the above reactions were carried out in triplicate.

### Carbaryl degradation by free CarCby and whole cell biocatalyst

High performance liquid chromatography (HPLC) (LC-MS-8045, SHIMADZU, Japan) and mass spectrometry (MS) equipped with an LC capillary column VP-ODS-C_18_ (2.0 × 150 mm, 5.0 μm) were employed to analyze carbaryl and its metabolites. 200 U of free CarCby or whole cell biocatalyst was incubated with 70 mg/L of carbaryl at 30 °C, pH 8.0 or 7.5, respectively. Samples were collected every 2 h and mixed with *n*-hexane and acetone (3:2, v/v), and extracts were obtained by ultrasonication for 15 min. Finally, the solvent was volatilized from the extracts at 37 °C and re-dissolved in methanol with an equal volume. The operation conditions were set at an initial temperature of 40 °C using a gradient of formic acid/acetonitrile at a flow rate of 0.4 mL/min, and 2 µL of each reaction extract was separately injected. Column elution was monitored by measuring the absorbance at 280 nm. The conditions for MS analysis were ionization at 4 kV, and the carbaryl or 1-naphthol fragment ions were detected by electrospray ionization with positive polarity.

### Accession number

The nucleotide sequence of *carCby* gene (1449 bp) was deposited in GenBank database under the accession number of OM336259.

## Supplementary Information

**Additional file 1: Fig. S1**. Phylogenetic analysis of CarCby with other carboxylesterases from different microorganisms.

**Additional file 2: Fig. S2**. SDS-PAGE analysis of CarCby mutants.

**Additional file 3: Table S1**. Kinetic parameters of recombinant CarCby and catalytic triads mutants at 30 °C.

**Additional file 4: Fig. S3**. Membrane sensitive to 2 mM EDTA.

**Additional file 5: Fig. S4**. Mass spectrum of the metabolites in the reaction.

**Additional file 6: Fig. S5**. Proposed metabolic pathway of carbaryl by B. velezensis sd.

**Additional file 7: Table S2**. Predicted proteins involved in carbaryl metabolism in B. velezensis sd.

**Additional file 8: Fig. S6**. Schematic diagram of different expressional plasmids construction.

## Data Availability

All data generated or analyzed during this study are included in this published article and its Additional files.
