# Supplementary material for: Display of a novel carboxylesterase CarCby on Escherichia coli cell surface for carbaryl pesticide bioremediation
Source: Microb Cell Fact. 2022 May 28;21:97. doi: 10.1186/s12934-022-01821-5 (PMC9148518; doi:10.1186/s12934-022-01821-5)
Supplement: Supplementary file 1 — Additional file 1: Fig. S1. Phylogenetic analysis of CarCby with other carboxylesterases from different microorganisms. [file 12934_2022_1821_MOESM1_ESM.docx]

**Supplementary Information**

**Display of a novel carboxylesterase CarCby on *Escherichia coli* cell surface for carbaryl pesticide bioremediation**

Yan Liu, Xiaoliang Wang, Sujin Nong, Zehui Bai, Nanyu Han, Qian Wu, Zunxi Huang, Junmei Ding^⚹^

Engineering Research Center of Sustainable Development and Utilization of Biomass Energy, Ministry of Education, Yunnan Normal University, Kunming, Yunnan, 650500, China

^⚹^Correspondence should be addressed to J.M. Ding: [djm3417@163.com](mailto:djm3417@163.com).

**Additional file 1: Fig. S1** Phylogenetic analysis of CarCby with other carboxylesterases from different microorganisms.

**Additional file 2: Fig. S2** SDS-PAGE analysis of CarCby mutants.

**Additional file 3: Table S1** Kinetic parameters of recombinant CarCby and catalytic triads mutants at 30 °C.

**Additional file 4: Fig. S3** Membrane sensitive to 2 mM EDTA.

**Additional file 5: Fig. S4** Mass spectrum of the metabolites in the reaction.

**Additional file 6: Fig. S5** Proposed metabolic pathway of carbaryl by *B. velezensis* sd.

**Additional file 7: Table S2** Predicted proteins involved in carbaryl metabolism in *B. velezensis* sd.

**Additional file 8: Fig. S6** Schematic diagram of different expressional plasmids construction.


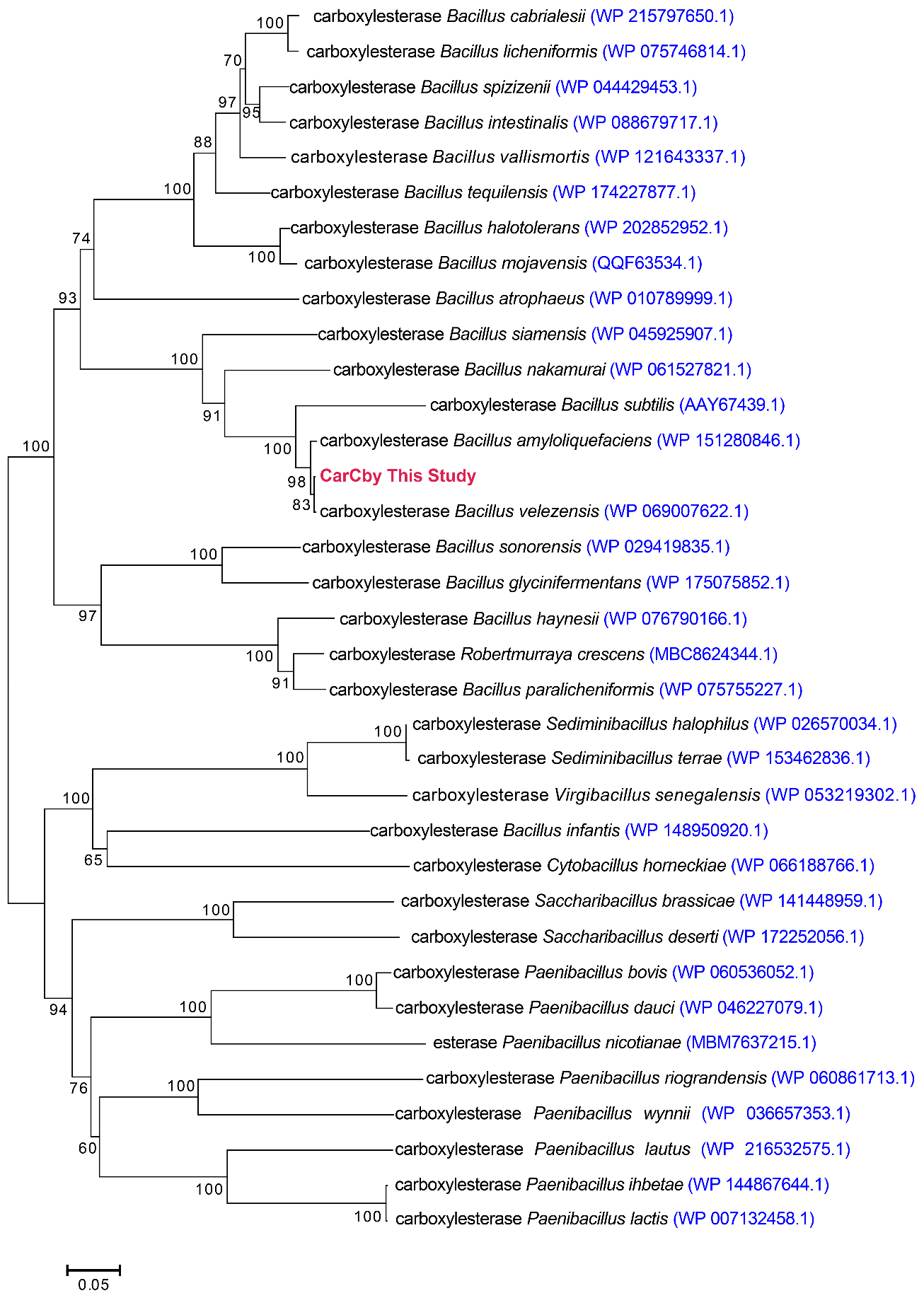


**Additional file 1: Fig. S1.** Phylogenetic analysis of CarCby with other carboxylesterases from different microorganisms. Sequences are obtained from GenBank and their source strains together with accession numbers are given behind
