# Supplementary material for: Display of a novel carboxylesterase CarCby on Escherichia coli cell surface for carbaryl pesticide bioremediation
Source: Microb Cell Fact. 2022 May 28;21:97. doi: 10.1186/s12934-022-01821-5 (PMC9148518; doi:10.1186/s12934-022-01821-5)
Supplement: Supplementary file 2 — Additional file 2: Fig. S2. SDS-PAGE analysis of CarCby mutants. [file 12934_2022_1821_MOESM2_ESM.docx]

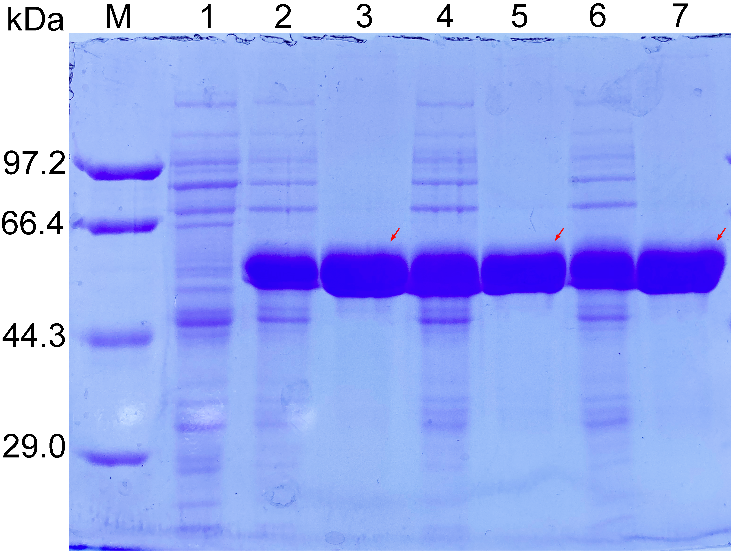


**Additional file 2: Fig. S2.** SDS-PAGE analysis of CarCby mutants. Lane M: protein marker; Lane 1: supernatant of *E. coli* BL21(DE3)[pET-28a(+)] strain (control) lysates; Line 2, 4, and 6: supernatant of *E.coli* BL21(DE3) containing pET-28a(+)/CarCbySer190A, pET-28a(+)/CarCbyGlu306A, pET-28a(+)/CarCbyHis395A lysates, respectively; Line 3, 5, and 7: purification of recombinant CarCbySer190A, CarCbyGlu306A, and CarCbyHis395A mutants, respectively. The recombinant mutants of CarCby proteins are marked with red arrows
