# Supplementary material for: Display of a novel carboxylesterase CarCby on Escherichia coli cell surface for carbaryl pesticide bioremediation
Source: Microb Cell Fact. 2022 May 28;21:97. doi: 10.1186/s12934-022-01821-5 (PMC9148518; doi:10.1186/s12934-022-01821-5)
Supplement: Supplementary file 4 — Additional file 4: Fig. S3. Membrane sensitive to 2 mM EDTA. [file 12934_2022_1821_MOESM4_ESM.docx]

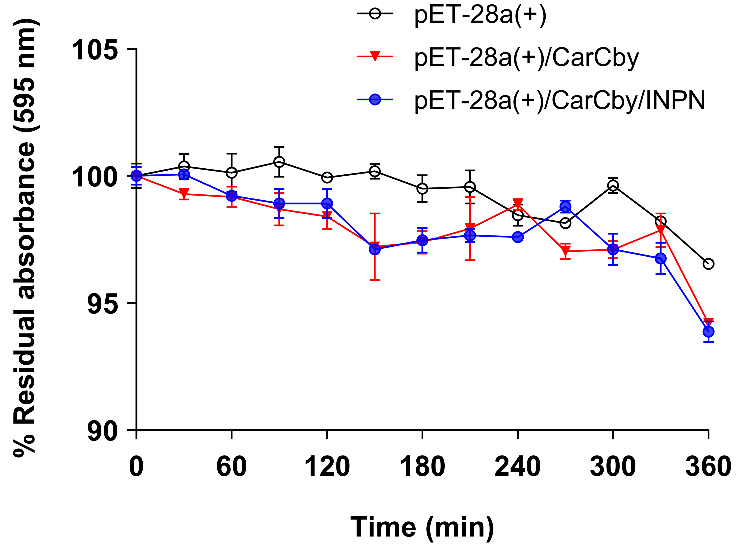


**Additional file 4: Fig. S3.** Membrane sensitive to 2 mM EDTA. The cell integrity of BL21(DE3) cells containing pET-28a(+)*/*CarCby*/*INPN and pET-28a(+)*/*CarCby, respectively, BL21(DE3) cells harboring pET-28a(+) blank vector was used as a control.
