# Supplementary material for: Display of a novel carboxylesterase CarCby on Escherichia coli cell surface for carbaryl pesticide bioremediation
Source: Microb Cell Fact. 2022 May 28;21:97. doi: 10.1186/s12934-022-01821-5 (PMC9148518; doi:10.1186/s12934-022-01821-5)
Supplement: Supplementary file 5 — Additional file 5: Fig. S4. Mass spectrum of the metabolites in the reaction. [file 12934_2022_1821_MOESM5_ESM.docx]

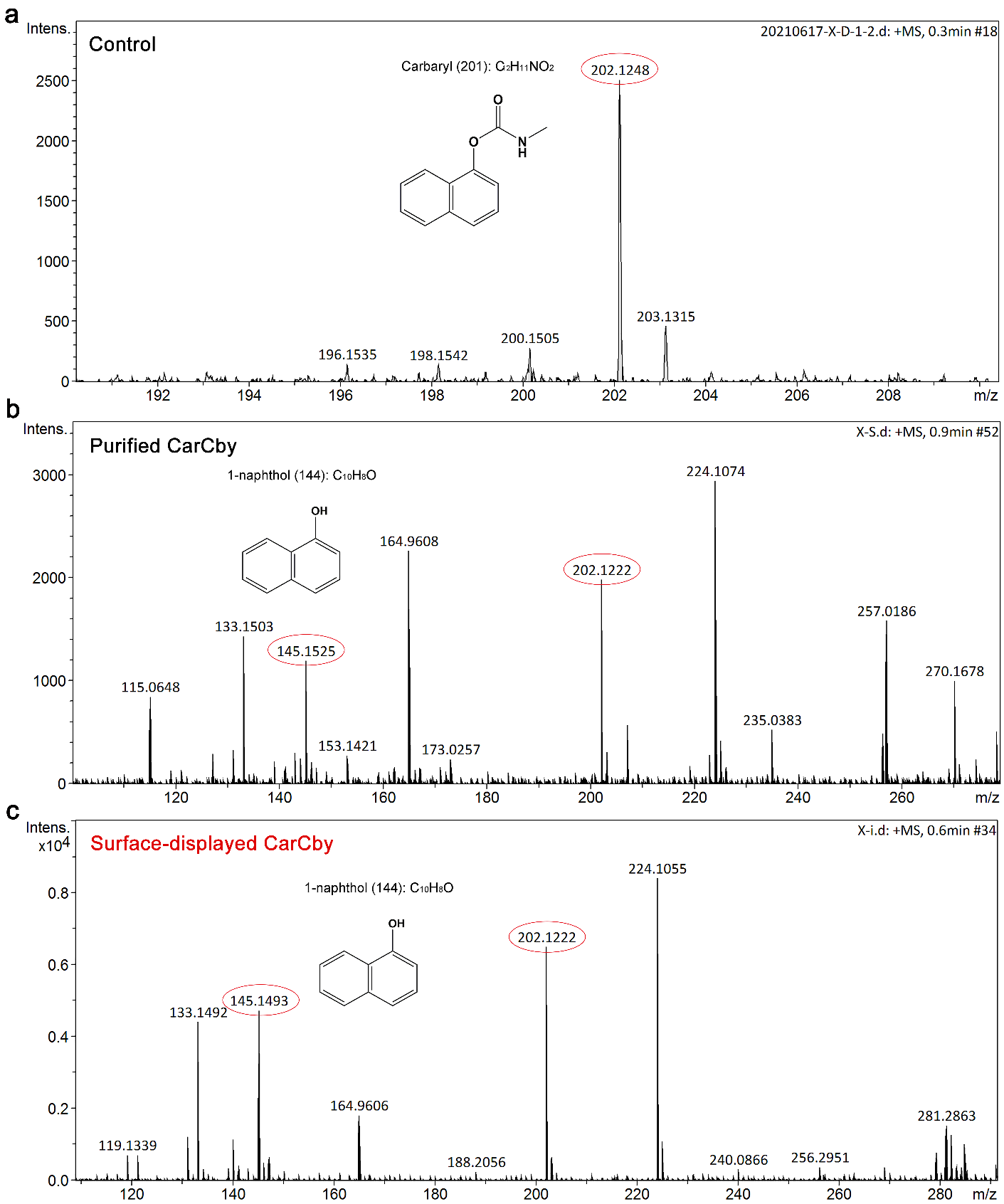


**Additional file 5: Fig. S4.** Mass spectrum of the metabolites in the reaction. **a** Carbaryl (control). **b** Reaction with carbaryl and purified CarCby. **c** Reaction with carbaryl and surface-displayed CarCby. 1-naphthol with an *m/z* [M+H]^+^ of 145 was detected in both reactions **b** and **c**
