# Supplementary material for: Display of a novel carboxylesterase CarCby on Escherichia coli cell surface for carbaryl pesticide bioremediation
Source: Microb Cell Fact. 2022 May 28;21:97. doi: 10.1186/s12934-022-01821-5 (PMC9148518; doi:10.1186/s12934-022-01821-5)
Supplement: Supplementary file 6 — Additional file 6: Fig. S5. Proposed metabolic pathway of carbaryl by B. velezensis sd. [file 12934_2022_1821_MOESM6_ESM.docx]

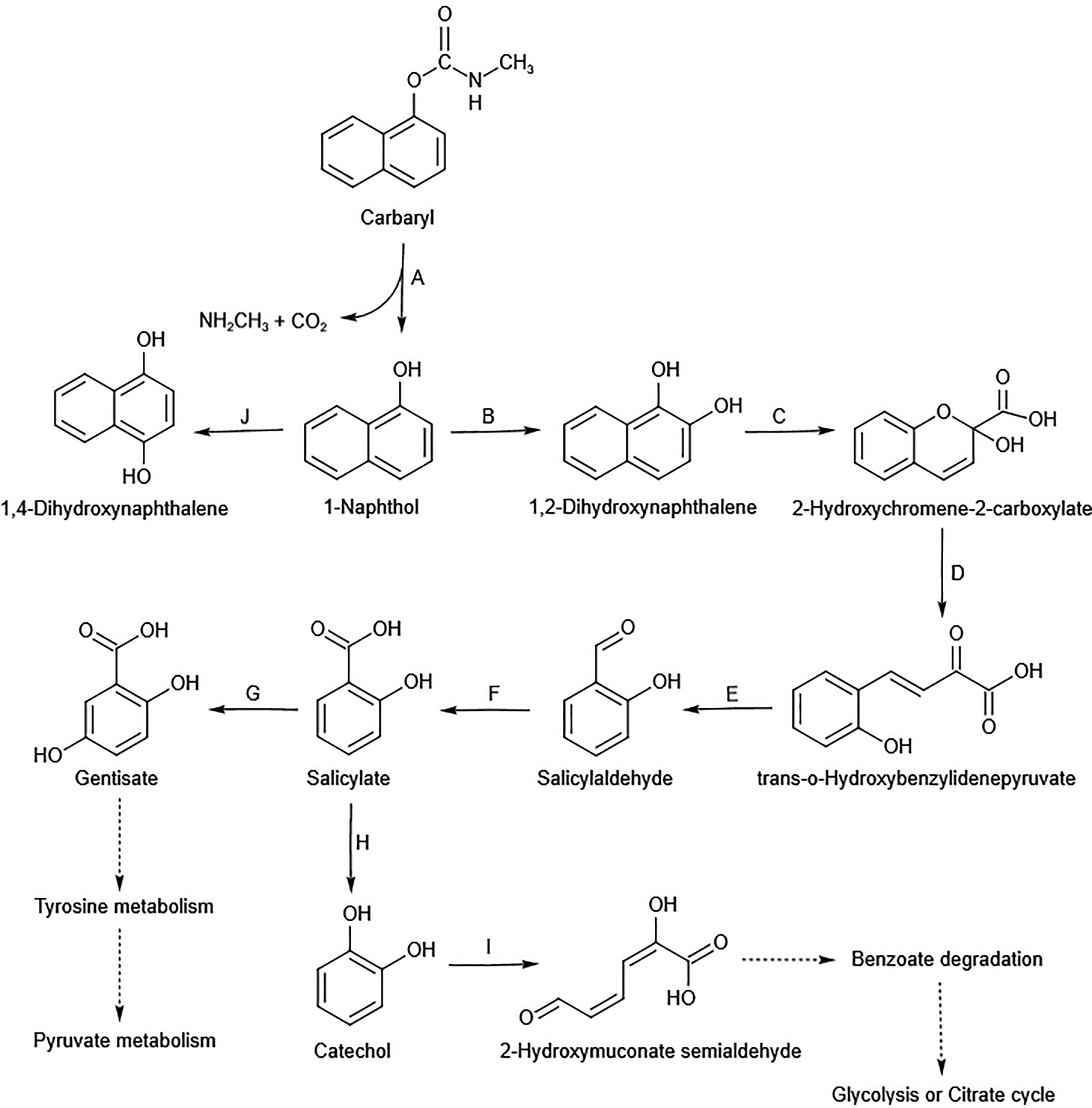


**Additional file 6: Fig. S5.** Proposed metabolic pathway of carbaryl by *B. velezensis* sd. A-J represent the predicted enzymes involved in each metabolic step
