# Supplementary material for: Display of a novel carboxylesterase CarCby on Escherichia coli cell surface for carbaryl pesticide bioremediation
Source: Microb Cell Fact. 2022 May 28;21:97. doi: 10.1186/s12934-022-01821-5 (PMC9148518; doi:10.1186/s12934-022-01821-5)
Supplement: Supplementary file 7 — Additional file 7: Table S2. Predicted proteins involved in carbaryl metabolism in B. velezensis sd. [file 12934_2022_1821_MOESM7_ESM.docx]

**Additional file 7: Table S2** Predicted proteins involved in carbaryl metabolism in *B. velezensis* sd

| **Steps** | **Predicted proteins** | **Location in genome** | **Protein homolog** | **Protein length (amino acid）** |
| --- | --- | --- | --- | --- |
| A | CarCby | 3331583-3333031 | Phenyl carbamate hydrolase (Q01470.1) | 483 |
| B | 4-hydroxyphenylacetate-3-monooxygenase | 232469-231021 | cehC1 (QRI93549.1) | 483 |
| C  D  E  F  G  H  I  J | Oxidoreductase  Uncharacterized protein YwbO  4-hydroxy-tetrahydrodipicolinate synthase  Aldehyde dehydrogenase  Nitrite reductase [NAD(P)H] small subunit  Salicylate hydroxylase  Catechol-2,3-dioxygenase  Cytochrome P450 102A3 | 1331973-1332719  3698748-3698146  1680708-1681577  732053-733510  321529-321209  716081-717202  836669-837526  718453-721656 | 1,2-dihydroxynaphthalene dioxygenase (AAA91577)  2-hydroxychromene-2-carboxylate isomerase (AAA66358)  trans-o-hydroxybenzylidenepyruvate hydratase-aldolase (AAD45417)  salicylaldehyde dehydrogenase (BAE92159)  salicylate 5-hydroxylase large subunit (AAD12607)  salicylate hydroxylase (AAA25897)  catechol 2,3-dioxygenase (AAA23353)  cytochrome P450 1A1 isoform 1 (NP_000490) | 249  201  290  486  107  374  286  1068 |
