# Supplementary material for: Display of a novel carboxylesterase CarCby on Escherichia coli cell surface for carbaryl pesticide bioremediation
Source: Microb Cell Fact. 2022 May 28;21:97. doi: 10.1186/s12934-022-01821-5 (PMC9148518; doi:10.1186/s12934-022-01821-5)
Supplement: Supplementary file 8 — Additional file 8: Fig. S6. Schematic diagram of different expressional plasmids construction. [file 12934_2022_1821_MOESM8_ESM.docx]

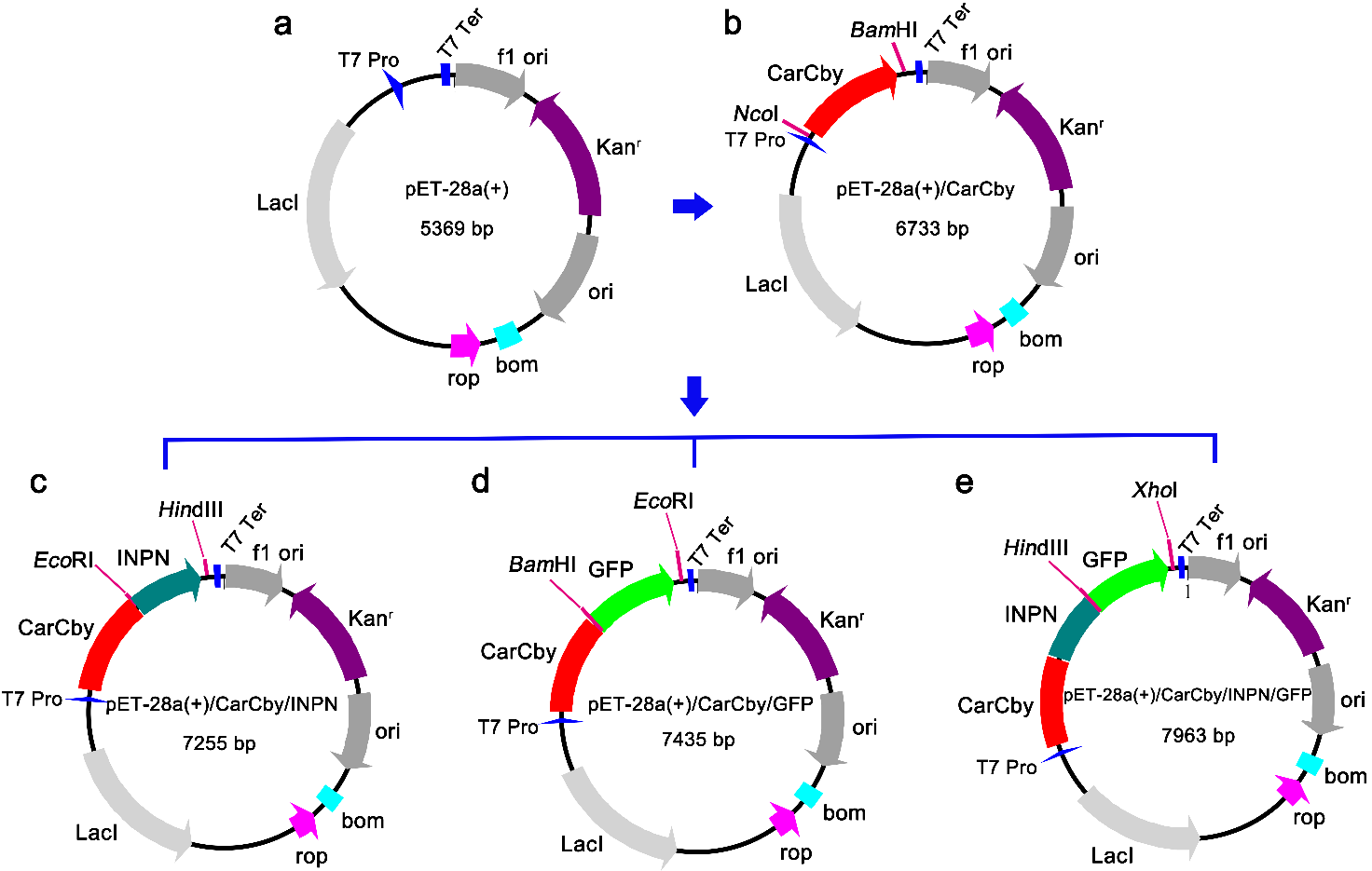


**Additional file 8: Fig. S6.** Schematic diagram of different expressional plasmids construction. **a** pET-28a(+). **b** pET-28a(+)/CarCby. **c** pET-28a(+)/CarCby*/*INPN. **d** pET-28a(+)/CarCby*/*GFP. (E) pET-28a(+)/CarCby*/*INPN*/*GFP
